# Aciclovir‐resistenter Zoster disseminatus bei chronischer lymphatischer B‐Zell‐Leukämie

**DOI:** 10.1111/ddg.15870_g

**Published:** 2026-01-14

**Authors:** Tabael L. Turan, Thomas K. Eigentler, Ulrike Blume‐Peytavi, Kamran Ghoreschi

**Affiliations:** ^1^ Klinik für Dermatologie Venerologie und Allergologie Charité – Universitätsmedizin Berlin Corporate Member of Freie Universität Berlin Humboldt‐Universität zu Berlin, and Berlin Institute of Health; ^2^ Berliner Institut für Gesundheitsforschung in der Charité – Universitätsmedizin Berlin BIH Charité Junior Clinician Scientist Programm

Sehr geehrte Herausgeber,

Patienten mit malignen hämatologischen Erkrankungen sind einem signifikant erhöhten Risiko ausgesetzt, einen Herpes zoster (HZ) zu entwickeln, also eine Reaktivierung latenter Varizella‐Zoster‐Viren (VZV). Potenziell tödliche HZ‐bedingte Komplikationen wie systemische VZV‐Dissemination treten bei diesen Patienten häufiger auf.^1^, ^2^ Bei Patienten mit chronischer lymphatischer Leukämie (CLL) sind sowohl die Erkrankung selbst als auch die vorgenommene Behandlung für eine fehlregulierte Immunantwort verantwortlich. Solche Patienten sind anfällig für Infektionen und Komplikationen, insbesondere in den ersten Jahren nach Diagnosestellung.^3^, ^4^ Die frühzeitige Einleitung wirksamer antiviraler Medikamente ist beim HZ prognostisch entscheidend, wobei intravenöses Aciclovir (ACV) das Mittel der Wahl ist.^5^ Resistenzen von VZV auf ACV wurden vorrangig bei immundefizienten Patienten beschrieben und beruhen üblicherweise auf Mutationen des viralen Thymidinkinase (TK)‐Gens.^6^ Thymidinkinase‐unabhängige Zweitlinientherapien mit Virostatika wie Foscarnet (FOS) oder Cidofovir haben sich in solchen Fällen vereinzelt als wirksam erwiesen.^7^, ^8^ Hier berichten wir über den Fall einer Patientin mit chronischer lymphatischer B‐Zell‐Leukämie (B‐CLL) unter abwartendem Beobachten, die trotz langfristiger antiviraler Therapie mit ACV und FOS einen progressiven, disseminierten HZ mit Lungenbeteiligung entwickelte.

Eine 60‐jährige Frau stellte sich im Juli 2024 in der Notaufnahme mit einer seit zwei Tagen bestehenden, schmerzhaften vesikulären Eruption vor, welche das Dermatom V1 links betraf. Die Patientin berichtete, dass dem Ausschlag ein dreitägiges lokales Kribbeln vorausgegangen sei. Die Patientin gab keine anderen Symptome an, wie zum Beispiel Sehstörungen, und verneinte, in der Vergangenheit einen ähnlichen Ausschlag gehabt zu haben. Sie hatte zuvor noch keine HZ‐Impfung erhalten. Aus der Anamnese ging hervor, dass bei ihr im Jahre 2013 eine B‐CLL [del(13q)] diagnostiziert worden war, für die sie 2015/16 sechs Zyklen einer Erstlinien‐Chemoimmuntherapie mit Bendamustin plus Rituximab erhalten hatte, gefolgt von abwartendem Beobachten. Zwanzig Tage vor Aufnahme wurden im Rahmen der Nachsorge im Blut Leukozyten (*white blood cell*, WBC) von 49,8 × 10^3^/µl und ein normwertiges C‐reaktives Protein (CRP) gemessen. Zu diesem Zeitpunkt lag keine Hypogammaglobulinämie vor (IgG: 11,93 g/l).

Bei der körperlichen Untersuchung ließen sich etwa 25 Vesikel außerhalb des primär befallenen Dermatoms finden, welche die Nackenregion, den vorderen Rumpf sowie die oberen Extremitäten betrafen. Mit Ausnahme eines moderaten ipsilateralen Lidödems wurden keine okulären Manifestationen festgestellt. Ebenso war keine Lymphadenopathie palpabel. Die Blutentnahme ergab eine erhöhte CRP‐Konzentration (81,3 mg/l) und eine relative Lymphozytose (51,3 %) mit Kernschatten (41,2 %). Die WBC (10,4 × 10^3^/µl) lagen innerhalb des Referenzbereiches, vereinbar mit einem signifikanten (sub‐)akuten Rückgang im Vergleich zu den prästationären Werten (Abbildung 1). Die Durchflusszytometrie des peripheren Blutes bestätigte einen B‐CLL‐Immunphänotyp (CD19‐Expression > 95 %).

**ABBILDUNG 1:**
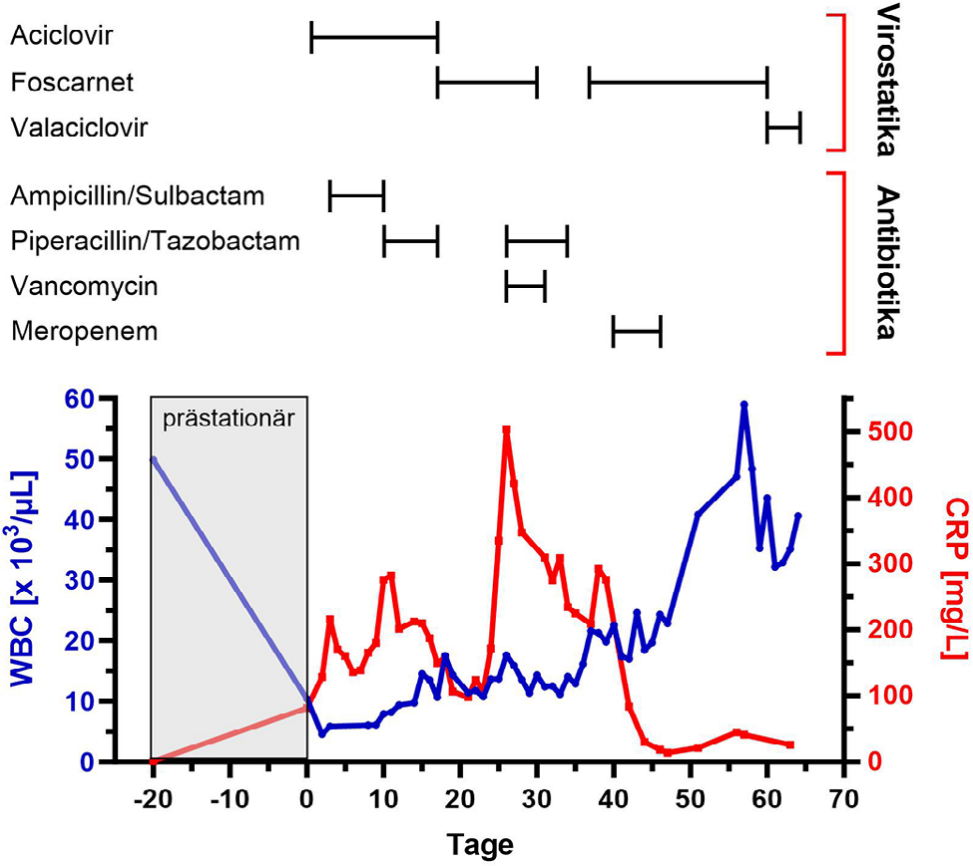
Zeitlicher Verlauf des C‐reaktiven Proteins (CRP; rote Linie) und der Leukozyten (WBC; blaue Linie) im Serum einer hospitalisierten Patientin mit disseminiertem Zoster und zugrunde liegender B‐CLL, bezogen auf die verabreichten Antiinfektiva.

Nach klinischer Diagnosestellung eines HZ ophthalmicus (HZO) mit kutaner Dissemination wurde die Patientin mit hochdosiertem intravenösem ACV (10 mg/kg KG 3 x täglich) behandelt. Da das CRP am dritten Tag des stationären Aufenthaltes auf 216,3 mg/l anstieg, ohne dass es klinische, laborchemische oder radiologische Zeichen eines zusätzlichen Infektfokus gab, wurde eine Antibiose mit Ampicillin/Sulbactam (2 g/1 g 3 x täglich) eingeleitet. Trotz eines zwischenzeitlichen Rückgangs stieg das CRP weiter an, sodass die Antibiose auf Piperacillin/Tazobactam (4 g/0,5 g 3 x täglich) eskaliert wurde. Danach sank das CRP signifikant, woraufhin die antibiotische Therapie bei fehlendem Nachweis einer bakteriellen Infektion an Tag 16 abgesetzt wurde. Klinisch wurde hingegen kein Ansprechen auf die antivirale Therapie festgestellt, da sich neue aberrierende Vesikel über große Teile des Integumentes ausbreiteten, hinweisend auf ACV‐resistente VZV. An Tag 14 wurde die Bläschenflüssigkeit mittels Polymerase‐Kettenreaktion hochpositiv auf VZV‐DNA getestet. Serologisch ließen sich im Enzymimmunoassay positive VZV‐IgG‐Antikörper nachweisen (2782 mIU/ml), während die VZV‐IgM‐ und VZV‐IgA‐Antikörper negativ waren.

An Tag 16 wurde eine Zweitlinientherapie mit FOS (40 mg/kg KG 3 x täglich) gestartet, die als intermittierende Infusion über einen peripher eingeführten zentralvenösen Katheter (PICC) verabreicht wurde. Das CRP im Serum sank daraufhin innerhalb von 5 Tagen um etwa 48 % und erreichte an Tag 21 einen Tiefststand von 97,7 mg/l. Gleichzeitig kam es zu geringer klinischer Besserung des zosteriformen Exanthems. Kurz darauf wurden jedoch CRP‐Konzentrationen > 300 mg/l gemessen und der Allgemeinzustand der Patientin verschlechterte sich rapide. Nach Entnahme von Blutkulturen, PICC‐Entfernung und Einleitung einer empirischen antibiotischen Therapie mit Piperacillin/Tazobactam plus Vancomycin (1 g 2 x tgl.), wurde die Patientin an Tag 27 mit einem Blutdruck von 138/76 mmHg, einer Herzfrequenz von 109/min, einer Sauerstoffsättigung von 61 % und einer Körpertemperatur von 38,5°C auf die Intensivstation verlegt. Eine Computertomographie (CT) des Thorax bei Aufnahme auf die Intensivstation zeigte eine pulmonalvenöse Stauung ohne Infiltrate. Die mikrobiologische Untersuchung ergab keine spezifischen pathogenen Mikroorganismen, sodass die Antibiose im weiteren Verlauf abgesetzt wurde. Da zu diesem Zeitpunkt alle Vesikel eingetrocknet waren, wurde die Therapie mit FOS nach 13 Tagen beendet. Obwohl die Entzündungsparameter eine rückläufige Tendenz zeigten, blieb die Patientin auf eine nichtinvasive Beatmung angewiesen. Bei der nachfolgenden Bronchoskopie an Tag 35 wurde VZV‐DNA im Sekret der bronchoalveolären Lavage nachgewiesen. Darüber hinaus zeigte eine erneute Thorax‐CT ausgedehnte, atypische, bipulmonale Infiltrate (Abbildung 2a,b). Entsprechend wurden intravenöses FOS und Meropenem (1 g 3 x tgl.) eingeleitet, worunter es zu einem deutlichen und anhaltenden Rückgang des CRP kam. Zeitgleich kehrten die WBC auf das prästationäre Niveau zurück. An Tag 42 erhielt die Patientin intravenöse Immunglobuline angesichts eines kombinierten Immunglobulinmangels (IgG: 6,28 g/l; IgA: 0,35 g/l; IgM: 0,22 g/l). Schließlich beobachteten wir eine Regredienz der pulmonalen Infiltrate im Thorax‐Röntgen und eine Besserung der respiratorischen Situation der Patientin. An Tag 61 wurde FOS durch orales Valaciclovir (500 mg 2 x täglich) zur VZV‐Prophylaxe ersetzt, doch erforderte die hierunter aufgetretene Nephrotoxizität das Absetzen der antiviralen Medikation.

**ABBILDUNG 2:**
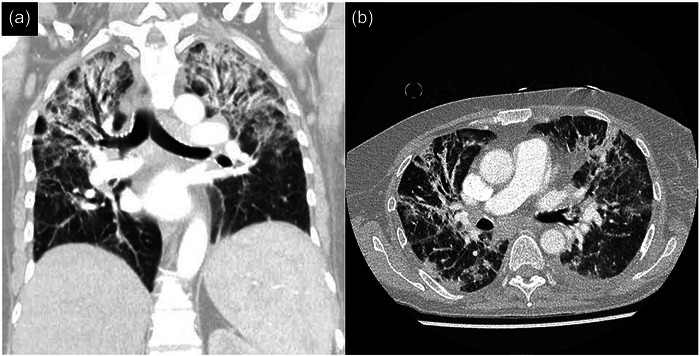
Computertomographie des Thorax in (a) koronarer und (b) axialer Schichtführung mit Nachweis bipulmonaler Oberlappeninfiltrate an Tag 38 des Krankenhausaufenthaltes.

Nach 65 Tagen Krankenhausaufenthalt wurde die Patientin klinisch stabil entlassen und erhielt einen Wiedervorstellungstermin bei den Hämatologen, um Zanubrutinib, einen Bruton‐Tyrosinkinase‐Inhibitor der nächsten Generation, als zielgerichtete Zweitlinientherapie bei CLL zu prüfen.

Die tiefgreifende Immundysregulation trägt zum erhöhten Infektionsrisiko bei CLL bei.^9^ Fälle protrahierter HZ‐Verläufe bei hämatologischen Patienten wurden bereits veröffentlicht.^6^, ^10^ Die Besonderheit dieses Fallberichts beruht auf zwei Aspekten: Zum einen trat ein schwerer HZ bei einer Patientin mit B‐CLL auf, die sich seit über 8 Jahren in regelmäßiger hämatologischer Kontrolle befand – also unabhängig vom Zeitpunkt der Erstdiagnose oder dem Beginn einer systemischen Therapie. Zum anderen handelt es sich um den ersten dokumentierten Fall, in dem eine Therapie mit Foscarnet bei einer B‐CLL‐Patientin mit Aciclovir‐refraktärem HZ erfolgreich eingesetzt wurde.

Die VZV‐Seroprävalenz > 90 % unter ungeimpften immunkompetenten Menschen bedingt ein hohes Risiko für die Entwicklung eines HZ.^11^ Der HZ mit viszeraler Dissemination ist in der Regel auf immunsupprimierte Patienten beschränkt, insbesondere jene mit aktiver Erkrankung oder kürzlicher Therapieeinleitung.^12^ Anhand einer Kohorte von CLL‐Patienten wurde beschrieben, dass VZV‐Reaktivierungen häufig und bis zu 3 Jahre nach Erstlinientherapie mit Bendamustin plus Rituximab auftreten, und zwar unabhängig von einer antiviralen Prophylaxe.^13^ Neben den ausgeprägten Alterationen der T‐Zell‐ und Neutrophilenfunktion gilt die Hypogammaglobulinämie als zentraler Faktor, der CLL‐Patienten für infektiöse Komplikationen prädisponiert.^9^, ^14^ Interessanterweise war bei unserer Patientin keiner dieser Risikofaktoren gegeben. Daher kann die VZV‐Reaktivierung in diesem Fall als Erstmanifestation eines Krankheitsprogresses interpretiert werden, die auf die mögliche Notwendigkeit einer Zweitlinientherapie im weiteren Verlauf hindeutet. In diesem Zusammenhang beobachteten wir einen passageren Rückgang der WBC während des Krankenhausaufenthaltes, der am ehesten auf einen gesteigerten Umsatz im Zuge der akuten viralen Infektion zurückzuführen ist.

Laut der *Deutschen Gesellschaft für Hämatologie und Medizinische Onkologie* (DGHO) benötigen Patienten mit malignen hämatologischen Erkrankungen bei disseminiertem HZ intravenöses ACV (10 mg/kg KG 3 x täglich) für mindestens 14 Tage.^15^ Eine Resistenz von VZV gegenüber ACV wurde in seltenen Fällen bei immundefizienten Patienten mit AIDS,^16^ hämatoonkologischen Erkrankungen^17^ oder nach hämatopoetischer Stammzelltransplantation nachgewiesen.^18^ Im vorliegenden Fall gab es kein suffizientes klinisches Ansprechen auf die empfohlene antivirale Therapie. Eine Resistenz gegenüber ACV hätte durch phänotypische oder genotypische Charakterisierung der VZV‐TK ermittelt werden können. Die genotypische Resistenzbestimmung in spezialisierten Laboren ist für den klinischen Einsatz aufgrund des geringeren Zeitaufwandes vorzuziehen. Allerdings ist die VZV‐Mutationsanalyse in Deutschland nicht allgemein verfügbar. Zudem kann eine klinische Resistenz auf ACV häufig nicht virologisch verifiziert werden, da die spezifischen VZV‐Mutationen, die eine antivirale Resistenz bewirken, noch nicht umfassend charakterisiert wurden.^17^, ^19^ Neben der Arzneimittelresistenz könnte auch eine unzureichende Dosierung von ACV zu dem klinischen Behandlungsversagen geführt haben. So erlangte beispielsweise ein 6‐jähriger Junge mit akuter lymphatischer Leukämie und VZV‐Enzephalitis, der mit 20 mg/kg KG intravenösem ACV 3 x täglich behandelt wurde, eine vollständige funktionelle Genesung.^20^ In Anbetracht dessen, dass ACV hauptsächlich renal ausgeschieden wird, könnte eine Dosiseskalation vor allem bei Patienten mit einer hohen glomerulären Filtrationsrate erforderlich sein.

Bemerkenswerterweise zeigte sich unter der Behandlung mit FOS ein biphasischer klinischer Verlauf, bei dem es zur vollständigen Regredienz des zosteriformen Exanthems kam, gefolgt von einer schweren VZV‐Pneumonie. Das Pausieren von FOS nach 13 Tagen könnte hierbei ein entscheidender Faktor gewesen sein. Dies bleibt jedoch spekulativ, da keine virologische Überwachung durchgeführt wurde und die vermutete ACV‐Resistenz der VZV‐Isolate nicht genotypisch nachgewiesen wurde. Nichtsdestotrotz sollte eine verlängerte Gabe von FOS über den Zeitpunkt hinaus, an dem alle Vesikel eingetrocknet sind, in künftigen Fällen von refraktärem disseminiertem HZ in Erwägung gezogen werden. Insgesamt stellt dieser seltene Fall eines HZO, der durch eine kutane und pulmonale Dissemination sowie eine klinische ACV‐Resistenz verkompliziert wurde, eine wertvolle Referenz für die klinische Praxis bei Patienten mit B‐CLL dar.

## DANKSAGUNG

Wir danken der Patientin für ihre Zustimmung, an dieser Studie teilzunehmen. Wir danken Herrn PD Dr. Alexander Lembcke und Herrn Dr. Dominik Deppe (Klinik für Radiologie, Charité – Universitätsmedizin Berlin) für die umfassende radiologische Beurteilung.

Dr. Turan nimmt am nationalen Translationalen Tandem‐Programm für gen‐ und zellbasierte Therapien (nTTP‐GCT) teil, das von der Biomedical Innovation Academy des Berlin Institute of Health at Charité (BIH) koordiniert und vom Bundesministerium für Bildung und Forschung (BMBF) gefördert wird.

Open access Veröffentlichung ermöglicht und organisiert durch Projekt DEAL.

## INTERESSENKONFLIKT

Keiner.
